# COVID-19 vaccines side effects among the general population during the pandemic: a cross-sectional study

**DOI:** 10.3389/fpubh.2025.1420291

**Published:** 2025-03-06

**Authors:** Samana Zaidi, Hafsa Abdul Qayyum, Izzah Abdul Qayyum, Zakir Khan, Taskeen Islam, Naveed Ahmed, Kathryn L. Hopkins, Theresa Sommers, Samar Akhtar, Shahzad Ali Khan, Sumbal Javed, Aamer Ikram, Hashaam Akhtar

**Affiliations:** ^1^Yusra Institute of Pharmaceutical Sciences, Yusra Medical and Dental College, Islamabad, Pakistan; ^2^School of Pharmacy and Biomolecular Sciences, Royal College of Surgeons (RCSI) University of Medicine and Health Sciences, Dublin, Ireland; ^3^Communication and Media Studies, Fatima Jinnah Women University, Rawalpindi, Pakistan; ^4^Department of Pharmacy, Quaid-i-Azam University, Islamabad, Pakistan; ^5^Sabin Vaccine Institute, Washington, DC, United States; ^6^Health Services Academy, Islamabad, Pakistan; ^7^Global Health Department, Health Services Academy, Islamabad, Pakistan; ^8^Armed Forces Institute of Pathology, Rawalpindi, Pakistan

**Keywords:** vaccine, precautionary medication, immunization, infodemic, side effects, COVID-19, Pakistan

## Abstract

**Background and aims:**

The general population have depicted concern about the safety and efficacy of the vaccine and its long-term effects on human health. Pakistan being on the verge of the pandemic is in more demand for vaccination and immunization. Therefore, this study aimed to evaluate the COVID-19 vaccines side effects among the general population.

**Methods:**

A cross-sectional face-to-face study was carried out among individuals who received either the first dosage or both doses of vaccination in twin cities (Islamabad and Rawalpindi) of Pakistan. Data was collected through a self-administered questionnaire. The questionnaire included three sections (socio-demographic, medical history, vaccine, and immunization) with 20 questions. The collected data was analyzed in SPSS (version 25) using descriptive statistics, the chi-square test, and the odd ratio.

**Results:**

A total of 2,618 participants were included and of them, females (55.3%; *n* = 1,449) were more than males. The majority of the participants reported the use of precautionary medicines including vitamin C (1,319; 50.4%) followed by paracetamol (*n* = 1,249; 47.7%) and mineral supplements (*n* = 616; 23.5%) for COVID-19. In this study, 3.8% (*n* = 99) were unvaccinated and the first and second doses of the vaccine was received by 2,519 and 2,239 of the participants, respectively. Different types of side effects were highlighted in the current study. The most frequently reported side effects after the first dose of COVID-19 were fever (*n* = 997), pain at the injection site (*n* = 994), muscle pain (*n* = 620), and fatigue (*n* = 482). Additionally, pain at the injection site (*n* = 852), fever (*n* = 815), and muscle pain (*n* = 601) were commonly reported after the 2nd dose of COVID-19. The lowest reported side effects were swollen lymph nodes and anaphylactic shock. In the current study, people who were previously immunized with the flu and pneumonia vaccine had a lower risk of developing side effects (*p* < 0.05).

**Conclusion:**

This study highlights important information about side effects reported due to the COVID-19 vaccinations. Moreover, the use of precautionary medications was also highlighted. These findings could have a valuable impact on designing future comparative studies and developing policies/guidelines for pandemic preparedness.

## Introduction

The new coronavirus COVID-19 is the most recent conflict in the series of pandemics, which originated in the Wuhan region of China in December 2019 (1, 2). It was not only spreading fear of a new virus but rather the chances of the resurgence of other known viruses, likewise Poliovirus increased dramatically, especially in low middle-income countries like Pakistan where the prevalence surged (3, 4). Most nations, including Pakistan, implemented strict precautionary measures to counter and control the spread of the epidemic, such as mandatory mask usage along quarantine approach work from home strategy, and smart lockdown (5–7). Being conducive to the halt of spread, these strategies are unable to ensure long-term protection and immunity against COVID-19 infection (8). Therefore, therapeutic, and preventative treatments seem to be paramount in managing COVID-19 infections (9).

Vaccines are considered to be a crucial step in controlling any disease. The development of COVID-19 vaccines was crucial and believed to be a vital weapon to fight the pandemic (10, 11). However, the World Health Organization (WHO) has listed vaccine hesitancy as one of the top 10 global health issues since 2019; it is fueled by false information about the efficiency and safety of vaccines (12, 13). A new challenge of misinformation and disinformation spread on social media platforms, which were later called as infodemic (10, 14). Side effects directly impact vaccine hesitancy and data from February 2022 shows that only 10.6% of the people from the global south compared to 61.9% of the World’s population received COVID-19 vaccine shots (15).

The Pakistani government has approved the following COVID-19 vaccines: the mRNA-based BNT162b2 (commercial name: Comirnaty, Pfizer—BioNTech); the inactivated virus-based BBIBP-CorV (commercial name: Covilo, BBIBP-CorV); CoronaVac (commercial name: CoronaVac, Sinovac); and the adenoviral vector-based ChAdOx1-S (commercial name: Vaxzevria, Oxford—AstraZeneca), Gam-COVID-Vac (commercial name: Sputnik V, Gamaleya), mRNA-1273 (commercial name: Spikevax, Moderna—NIAID) and AD5-nCOV (commercial name: Convidecia, CanSino). The COVID-19 vaccination recipients reported injection site or local reactions more commonly than systemic reactions and significant adverse effects were uncommon, according to global safety studies on vaccine reactogenicity (16). Females and the younger population group are more prone to report more adverse effects than the other groups (17, 18). A Plethora of people around the globe expressed their concerns and reluctance to get vaccinated despite published safety data on COVID-19 vaccines, and studies have revealed a correlation between vaccination intention and favorable vaccination attitudes (19).

The most common reason for vaccine hesitation among various population groups is an aversion to the potential negative effects of immunizations (20). Similarly, a recent systematic analysis found that increasing the public’s understanding of vaccines’ effectiveness, and honesty regarding their side effects, are vital strategies to improve vaccine uptake (21). The current study is related to the assessment of the COVID-19 vaccine’s side effects and acceptance amongst the Pakistani population. This study aims to collect data through the quantitative methodology to assess the type and frequency of side effects experienced by individuals after being administered 1st and 2nd dose of COVID-19 vaccines, respectively. This study also intends to configure which vaccines were preferred by individuals in twin cities and which side effects were most prevalent. This knowledge would also give people more confidence to acquire COVID-19 vaccinations, hence increasing vaccine acceptance. Therefore, this study aimed to investigate the COVID-19 vaccine’s side effects and acceptance among the general population in the Twin Cities (Rawalpindi and Islamabad) of Pakistan.

## Materials and methods

### Study design and setting

A 6-month cross-sectional face-to-face questionnaire-based study was carried out among the general population in Twin cities (Rawalpindi and Islamabad) of Pakistan. Islamabad-Rawalpindi is the fourth-largest metropolitan area in Pakistan.

### Inclusion and exclusion criteria

This study included the general population with age ≤ 20 years, residing in selected study settings, and vaccination status. The individuals who did not belong to the Islamabad/ Rawalpindi areas and were non-consented to participate were excluded from this study.

### Sample size

According to the World Population Review, the population of Islamabad and Rawalpindi was 1,163,580 and 2,280,730 in 2021, respectively (22, 23). The total population was 3,525,490. This information was entered into the Raosoft sample size calculator and a minimum required sample size was 385 with an assumed 50% response distribution, 5% error margin, and 95% confidence level (24). We distributed a questionnaire with 2,650 participants to provide a broad perspective, avoid missing data, and account for the response rate.

### Data collection method and study tool (questionnaire)

A self-administered questionnaire was prepared through a literature search (16–18, 21). Initially, the questionnaires were prepared in English and translated into the Pakistan national language (Urdu) then back to the English language to ensure consistency. Three academic expert researchers then examined the questionnaire to assess its validity, appropriateness, consistency, and adequacy. The questionnaire was tested with 20 participants at the Yusra Institute of Pharmaceutical Sciences (YIPS), Islamabad, and Rawalpindi Medical University, Rawalpindi, Pakistan to verify the conception of the questionnaire’s language and suitability for reliably measuring the variables under observation. Participants in the pilot study were excluded from the final sample. Minor modifications were made following the suggestions. Finally, the questionnaire was distributed among eligible participants visiting COVID-19 vaccination camps in various hospital settings in Islamabad and Rawalpindi. The data for this study was gathered for 8 months starting from September 1, 2020, to April 30, 2021.

The final questionnaire comprised 3 core sections with 20 questions (Supplementary Data Sheet 1). The first section was about the socio-demographic characteristics (sex, age, marital status, education, employment, and residence). The second section included medical history. The third section contained vaccines and immunization-related questions (previous immunization status, COVID-19 test, results, vaccination status against COVID-19, type of vaccine, dose status of vaccine, and side effects after the first and second dose of COVID-19).

### Statistical analysis

Descriptive statistics were utilized to assess the participant’s data. Data was analyzed using Microsoft Excel (Microsoft Corp,) and IBM-SPSS version 25 (Chicago, Illinois, U.S.A.). All of the quantitative variables in the study were presented in the form of frequencies and percentages. The chi-square test and odd ratio were also used for analysis purposes. Statistical significance is defined as a *p*-value of less than 0.05.

## Results

### Sociodemographic information

This study involved a total of 2,618 participants, and among them, 44% were male (*n* = 1,169) and 55.3% were female (*n* = 1,449). Of them, 34.6% of the participants were ≤ 20 years of age, 56.8% of participants were grouped in the age group of 21–40 years, and 8.6% of participants were grouped in the ≥41 year age group. Most of the participants had bachelor-level degrees (58.1%), followed by post-graduates (22.8%), intermediate (5.7%), and matriculation (13.4%). About, 68.6% of the study participants were employed while 23.9% of the participants were unemployed (Table 1).

**Table 1:** Demographic information of the respondents.

Characteristics			
Variables	Values (V)	Number (*N*) *N* = 2,618	Percent (%)
Gender	Male	1,169	(44.7%)
	Female	1,449	(55.3%)
Age	≤ 20	906	(34.6%)
	21–40	1,486	(56.8%)
	≥ 41	226	(8.6%)
Education	Matriculation & below	352	(13.4%)
	Intermediate	149	(5.7%)
	Under-Graduate	152	(58.1%)
	Post-Graduate	596	(22.8%)
Occupational status	Employed	1795	(68.6%)
	Unemployed	627	(23.9%)
	Other	196	(7.5%)

### Types of precautionary medicines

**Figure 1:**
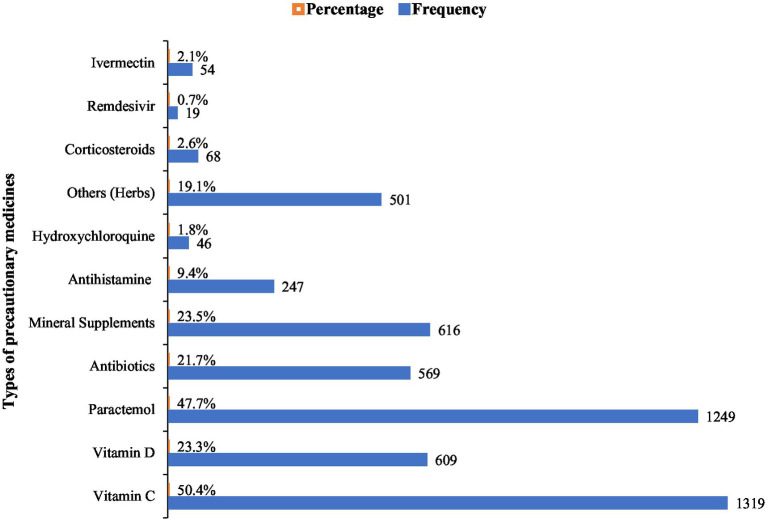
Types of precautionary medicines used for COVID-19.

The majority of the participants reported the use of precautionary medicines for COVID-19 including vitamin C, vitamin D, analgesic antipyretic drugs (paracetamol), antibiotics (macrolides, cephalosporins, and fluoroquinolones groups), mineral supplements (zinc, magnesium, calcium, iron, selenium), herbs, anti-allergic (antihistamine), hydroxychloroquine, corticosteroids, redeliver and ivermectin. Among them, vitamins showed the highest frequency (50.4%) followed by paracetamol (47.7%), mineral supplements (23.5%), and vitamin D (23.3%). While remdesivir and hydroxychloroquine showed the lowest frequency (Figure 1).

### Types of vaccines

**Figure 2:**
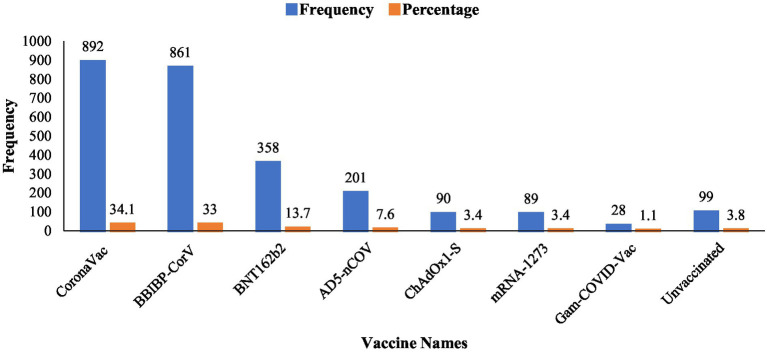
Types of vaccines.

Different types of vaccines were analyzed in this study, including BBIBP-CorV, CoronaVac, ChAdOx1-S, BNT162b2, AD5-nCOV, mRNA-1273, and Gam-COVID-Vac. Among them, CoronaVac showed the highest frequency (*n* = 892; 34.1%), followed by BBIBP-CorV (*n* = 861; 33%). Meanwhile, mRNA-1273 and ChAdOx1-S had a low usage rate of only 3.4%. Lastly, Gam-COVID-Vac had the lowest frequency (*n* = 28; 1.1%). Moreover, ninety-nine (*n* = 99; 3.8%) participants in this study were unvaccinated (Figure 2).

### Trend of side effects after the first and second doses of vaccines

This study also determined several types of side effects of vaccines after the first and second vaccine doses. Fever, Pain at the injection site, muscle pain, fatigue, itching, redness and swelling, headache, joint pain, cough, nausea/vomiting, diarrhea, tingling, shortness of breath, swollen lymph nodes, and anaphylactic reaction were the common symptoms that reported by the participants. Among them, after the first dose of the vaccine, fever showed the highest frequency (*n* = 997; 39.57%) while after the second dose of the vaccine, pain at the injection site showed the highest frequency (*n* = 852; 38%). Swollen lymph nodes and anaphylactic shock showed the lowest frequency (*n* = 11; 0.4%) after the first dosage of vaccine, while after the second dose of vaccine anaphylactic shock showed the lowest frequency (*n* = 6; 0.26%) (Table 2).

**Table 2:** The trend of side effects after first and second doses of vaccines.

Side effects of COVID-19 vaccines	After 1st dose		After 2nd dose	
Symptoms	Number of participants	Percentage frequency	Number of participants	Percentage frequency
Symptoms	*N* (2519)	(%)	*N* (2239)	(%)
No Symptoms	362	14.37	355	15.8
Fever	997	39.57	815	36.4
Pain at the injection site	994	39.46	852	38.0
Muscle pain	620	24.61	601	26.8
Fatigue	482	19.13	409	18.2
Itching, redness, and swelling	463	18.38	365	16.3
Headache	456	18.10	405	18.0
Joint pain	75	2.97	59	2.63
Cough	50	1.98	26	1.16
Nausea/Vomit	46	1.82	35	1.56
Diarrhea	28	1.11	21	0.9
Tingling	26	1.0	10	0.44
Shortness of breath	15	0.6	13	0.58
Swollen lymph nodes	11	0.4	7	0.31
Anaphylactic reaction	11	0.4	6	0.26

### Relationship between COVID-19 vaccine-related side effects and previously immunized participants with flu and pneumonia vaccine

The present study has also analyzed and compared the previously immunized participants with the flu vaccine and pneumonia vaccine in relationship to their side effects after the first and second doses of COVID-19 vaccines. The has revealed that the people who were previously immunized with the flu vaccine had a lower risk of developing tingling (*p*-value = 0.00), fever (*p*-value = 0.01), pain at the injection site (p-value = 0.01) and anaphylactic shock (*p*-value = 0.02) after the first dose of the vaccine because the p-value is lower than 1% as shown in Table 3. The participants immunized with the flu vaccine also reported a lower chance of developing headache (*p*-value = 0.02), joint pain (*p*-value = 0.05), diarrhea (*p*-value = 0.00), swelling, itching, and redness at the injection site (*p*-value = 0.04) after the second dose of the vaccine.

**Table 3:** Relationship between COVID-19 vaccine-related side effects and previously immunized participants with flu and pneumonia vaccine.

Covariates	1st dose of COVID-19 vaccine		Covariates	2nd dose of COVID-19 vaccine	
	Odds ratio (95% CI)	*p* value		Odds ratio (95% CI)	*p* value
Previously immunized participants with flu vaccine					
Tingling	1.019 (0.453–1.096)	0.00	Headache	0.981 (0.784–1.227)	0.02
Fever	1.008 (0.854–1.191)	0.01	Joint pain	1.065 (0.621–1.827)	0.05
Pain at the injection site	1.009 (0.854–1.191)	0.01	Diarrhea	0.962 (0.387–2.393)	0.00
Anaphylactic shock	1.101 (0.321–3.769)	0.02	Swelling, itching, and redness at the injection site	0.975 (0.772–1.232)	0.04
Previously immunized participants with the pneumonia vaccine					
Fatigue	1.023 (0.835–1.252)	0.04	No Symptoms	0.979 (0.822–1.166)	0.05
Joint pain	0.978 (0.610–1.566)	0.00	Joint pain	1.066 (0.630–1.802)	0.05
Anaphylactic shock	0.886 (0.259–3.035)	0.03	Nausea/Vomiting	0.916 (0.459–1.826)	0.06
Swollen lymph nodes	0.886 (0.259–3.035)	0.03	Swollen lymph nodes	1.164 (0.260–5.213)	0.04

Similarly, the participants who were previously immunized with the pneumonia vaccine had a lower risk of developing fatigue (*p*-value = 0.04), joint pain (*p*-value = 0.00), anaphylactic shock (*p*-value = 0.03) and swollen lymph nodes (*p*-value = 0.03) after the first dose of the as shown in Table 3, and also such people had a lower chance of developing joint pain (*p*-value = 0.05), nausea and vomiting (*p*-value = 0.06), swollen lymph nodes (*p*-value = 0.04) after second dose of vaccine.

### Period of COVID-19 vaccine side effects

This study also investigated the span of side effects linked with several COVID-19 vaccinations, which focus on the period in which they affect. These findings were made about a variety of COVID-19 vaccines. It has been showed that both BBIBP-CorV and CoronaVac vaccines have higher percentages for vaccine-related side effects that were prolonged for 25 to 72 h with percentage values of 52.26 and 47.53%, respectively, while this value was lowest (32.14%) for Gam-COVID-Vac as shown in Table 4.

**Table 4:** Time period of COVID-19 vaccines side effects.

Time period of COVID-19 vaccines side effects (*n* = 2,618)	BBIBP-CorV *N* = 861 Respondents (R) Percent 33%		CoronaVac *N* = 892 Respondents (R) Percent 34.1%		ChAdOx1-S *N* = 90 Respondents (R) Percent 3.4%		BNT162b2 *N* = 358 Respondents (R) Percent 13.7%		AD5-nCOV *N* = 201 Respondents (R) Percent 7.6%	mRNA-1273 *N* = 89 Respondents (R) Percent 3.4%			Gam-COVID-Vac *N* = 28 Respondents (R) Percent 1.1%	
Percent %	R	% = R/N	R	% = R/N	R	% = R/N	R	% = R/N	R	% = R/N	R	% = R/N	R	% = R/N
None	132	15.33	143	16.03	6	6.66	40	11.17	29	14.43	4	4.49	3	10.71
≤ 24 h	242	28.10	282	31.61	22	24.44	71	19.83	65	32.3	21	23.6	9	32.14
25–72 h	450	52.26	424	47.53	54	60	213	59.49	97	48.26	56	62.9	9	32.14
> 72 h	37	4.29	43	4.82	8	8.8	34	9.49	10	4.97	8	8.98	7	25

## Discussion

The present study evaluated the side effects and acceptance of COVID-19 vaccines among the Pakistani population in the Rawalpindi/Islamabad regions. Studies have shown that the side effects which are associated with vaccines are a major problem resulting in vaccine hesitancy (25). Therefore, to achieve the maximum acceptance of any vaccine, its adverse side effects should be tackled effectively (26).

In this study, most of the participants took precautionary medicines to avoid vaccination including immunity-boosting vitamins and minerals supplements, paracetamol, antibiotics, antiviral and antiparasitic drugs, steroids, anti-allergic, and other herbal remedies. This depicts that most of the participants were aware of the disease-preventive role of vitamin C. Several studies have reported that vitamin C is critical in boosting the adaptive immune response of the body (27). Several studies have reported that vitamin C plays a major role in preventing COVID-19 infection, and its progression, and significantly reduced COVID-related mortality rates (28). About, 57% of the participants took only one precautionary medicine, while only 3 participants out of 2,618 took all 11 COVID-preventive medications. Studies have reported that taking multiple medications at the same time can lead to adverse side effects due to the chemical interaction of the drugs (29, 30). Therefore, it is important to be cautious while taking such multiple medication regimens. Moreover, it is also reported in prior studies that social media including online health literacy information was used during COVID-19. Such practice leads to COVID-19 misinformation and malpractices (31, 32). Therefore, interdisciplinary, multilevel approaches are needed to involve government and public health organizations and social media companies to control and prevent misinformation and promote better public health (31).

It was reported in this study that most of the sample population were vaccinated individuals. Both BBIBP-CorV and CoronaVac were the most frequently selected vaccines, while Gam-COVID-Vac was the least selected. It was also revealed that only 4% of the study participants were unvaccinated individuals. This data shows that vaccine acceptance was higher in the participants. Several studies have reported that vaccine acceptance is associated with awareness and knowledge of individuals about the disease and its vaccination (33). Furthermore, the acceptance of any particular vaccination is also directly associated with the strong acceptance of the vaccine (34). It is recommended that easy vaccination techniques that are comfortable for the public and enhanced interactions between patients or caregivers and healthcare staff are useful strategies for increasing vaccine acceptability (35).

This study also analyzed different side effects that were reported after the first and second doses of vaccines. Fever, pain at the injection site, muscle pain, fatigue, itching, redness and swelling, headache, joint pain, cough, nausea/vomiting, diarrhea, tingling, shortness of breath, swollen lymph nodes and anaphylactic reaction among them; pain at injection site showed the highest percentage (37.16%) and cough showed the lowest percentage (1.16%) after first dose of BBIBP-CorV. Fever showed the highest percentage (34.35%), and cough showed the lowest percentage (1.01%) after the second dose of the BBIBP-CorV. Pain at the injection site showed the highest percentage (41.14%) and cough showed the lowest percentage (1.79%) after the first dose of the CoronaVac vaccine. Fever showed the highest percentage (30.17%), and cough showed the lowest percentage (1.21%) after the second dose of the CoronaVac vaccine. Fever showed the highest percentage (47.7%) and cough showed the lowest percentage (4.44%) after the first dose of the ChAdOx1-S vaccine. Fever showed the highest percentage (48.23%) and cough showed the lowest percentage (1.17%) after the second dose of the ChAdOx1-S vaccine. Pain at the injection site showed the highest percentage (45.53%) and cough and joint pain showed the lowest percentage (2.23%) after the first dose of the BNT162b2 vaccine. Fever showed the highest percentage (43.15%) and cough showed the lowest percentage (0.29%) after the second dose of the BNT162b2 vaccine. Fever showed the highest percentage (65.16%) and cough showed the lowest percentage (3.37%) after the first dose of the mRNA-1273 vaccine. Fever showed the highest percentage (65.47%), and cough showed the lowest percentage (1.19%) after the second dose of the mRNA-1273 vaccine. Fever showed the highest percentage (53.57%), no symptoms (3.57%), cough (10.71%), and joint pain (0%) after the first dose of the Gam-COVID-Vac. Fever showed the highest percentage (42.30%), and cough showed the lowest percentage (3.84%) after the second dose of Gam-COVID-Vac. Data from several studies have reported that the COVID-19 vaccines, like other vaccines, can have some side effects including myalgia, malaise, fever, and headache and these symptoms generally resolve within a few days without causing any severe damage to the body (36, 37).

The majority of adverse reactions lasting for 25 to 72 h were for BBIBP-CorV (52.26%) and CoronaVac (47.53%) vaccinations, respectively, while symptoms persisting for more than 72 h were 4.29 and 4.82%. Similar trends were observed with the ChAdOx1-S and BNT162b2 vaccines, with adverse effects lasting 25 to 72 h being the most common (60 and 59.49%, respectively), and those lasting more than 72 h being the least common (8.8 and 9.49%). These data highlight the temporal heterogeneity of side effects among COVID-19 vaccinations, allowing for a deeper comprehension of the immunogenic features of these vaccines.

### Limitation and strength

Like every study, this study also had some limitations. Causal inferences cannot be made due to the cross-sectional nature of the study. The study only included participants from the Islamabad-Rawalpindi of Pakistan, limiting the findings’ generalizability. However, Islamabad-Rawalpindi is the fourth-largest resident area in Pakistan. Thus, it is expected that the outcomes in the other areas would not differ considerably. The current study also depends on self-reported data, which is susceptible to bias. However, despite this limitation, we believe that our findings are sound and provide baseline data that will assist healthcare policymakers and researchers in the future. This is the first study in Pakistan with a significant sample size on the side effects of COVID-19 vaccinations in the general population. This study also highlighted the need for multicenter studies in another region of Pakistan to further explore the COVID-19 side effects scenario.

## Conclusion

This study provided data about the side effects experienced by individuals in twin cities of Pakistan after being administered with the first and second doses of COVID-19 vaccines. It is concluded that the most prevalent side effects experienced were pyrexia or fever followed by headache and chills. CoronaVac was the most common choice for vaccine followed by BBIBP-CorV and ChAdOx1-S. The most prevalent precautionary medicines that were taken by individuals to minimize or avoid COVID-19 infection were vitamins and paracetamol. The longest time for a side effect to be present was 24–48 h. Therefore, it is concluded that the vaccine’s side effects are mild to moderate, and it is safe to administer and take multiple doses of COVID-19 vaccine to prevent the disease from spreading and to develop immunity from the virus. However, there is a need to perform further studies on larger populations that can cross-verify the consistency of these results.

## Data Availability

The original contributions presented in the study are included in the article/Supplementary material, further inquiries can be directed to the corresponding author.
