# Supplementary material for: COVID-19 vaccines side effects among the general population during the pandemic: a cross-sectional study
Source: Front Public Health. 2025 Mar 6;13:1420291. doi: 10.3389/fpubh.2025.1420291 (PMC11922928; doi:10.3389/fpubh.2025.1420291)
Supplement: Supplementary file 1 [file Data_Sheet_1.PDF]

Questionnaire in English and Urdu.

**SOCIODEMOGRAPHIC**	
آبادیاتی معلومات	
**Gender**	☐ Male ☐ Female
صنف	☐ مر ☐ عورت
**Age (years)**	
عمر (سال)	
**Marital status**	☐ Single ☐ Married ☐ Others
ازدواجی حیثیت	☐ واحد ☐ شادی شدہ ☐ دوسرے
**Education (Degree)**	
تعلیم (ڈگری)	
**Employment**	☐ Employed ☐ Unemployed ☐ Retired
روزگار	☐ ملازمت والا ☐ بے روزگار ☐ ریٹائرڈ
**Nationality**	☐ Pakistani ☐ Non-Pakistani
قومیت	☐ پاکستانی ☐ غیر پاکستانی
**Residence**	☐ Islamabad ☐ Rawalpindi
رہائش	☐ اسلام آباد ☐ راولپنڈی
**Medical History**	
طبی تاریخ	
**Do you suffer from any chronic diseases?**	☐ Yes ☐ No
کیا آپ دائمی بیماریوں میں مبتلا ہیں؟	☐ ہاں ☐ نہیں
**Which type of disease are you suffering from?**	☐
آپ کس قسم کی بیماری میں مبتلا ہیں؟	☐
**Smoking status**	☐ **Current smoker** ☐ **Ex-smoker** ☐ **Not a smoker**
تمباکو نوشی کی حیثیت	☐ موجودہ سگریٹ نوشی ☐ سابق تمباکو نوشی

☐ سگریٹ نوشی نہیں	
**Childhood illness**	☐ Measles ☐Mumps ☐Rubella<br>☐Chicken pox ☐Rheumatic fever ☐Polio<br>☐Other
☐خسرہ ☐ممپس ☐روبیلا ☐چکن پاکس<br>☐رمیٹی بخار ☐پولیو ☐دیگر	بچپن کی بیماری
**Vaccines and Immunization**	
ویکسین اور حفاظتی ٹیکے	
**Previous Immunization**	☐Tetanus ☐Hepatitis ☐Influenza<br>☐Pneumonia ☐Chicken pox ☐ MMR (Measles, Mumps, Rubella)
☐تشنج ☐ہیپاٹائٹ ☐انفلوئنزا ☐نمونیا<br>☐ چکن پاکس ☐ (خسرہ ، ممپس ، روبیلا)	حفاظتی ٹیکہ
**Did you test for COVID -19?**	☐**Yes** ☐**No**
☐ ہاں ☐نہیں	کیا آپ نے کویوڈ -19 کے لئے ٹیسٹ کیا؟
**What was the result?**	☐ Positive ☐Negative ☐Not tested
☐ مثبت ☐ منفی ☐تجربہ نہیں کیا	نتیجہ کیا نکلا؟
**Did you receive any vaccine against COVID 19?**	☐**Yes** ☐**No**
☐ہاں ☐ نہیں	کیا آپ کو کوڈ 19 کے خلاف کوئی ویکسین موصول ہوئی ہے؟
**Types of precautionary medicines:____________**	
**Which type of vaccine did you receive?**	☐SinoPharm ☐SinoVac ☐Oxford AstraZeneca<br>☐Pfizer-BioNTech ☐Sputnik V<br>☐ Moderna ☐Not vaccinated ☐Not known
☐سینوفارم ☐سینوواک ☐آکسفورڈ آسٹرا زینیکا<br>☐فائزر بائیو ٹیک ☐سپوتنک وی ☐ موڈرنا ویکسین<br>☐نہیں لگائی گئی ☐معلوم نہیں	آپ کو کس قسم کی ویکسین ملی ہے؟

**Did you receive both doses of vaccine?**	☐Only 1st dose ☐Both doses<br>☐Not vaccinated
☐ صرف 1 خوراک ☐دونوں خوراکیں ☐ ٹیکہ نہیں لگایا	کیا آپ کو ویکسین کی دونوں خوراکیں موصول ہوئی ہیں؟
**Where did you vaccinated from?**	☐Public setup ☐ Government setup<br>☐ Private setup ☐None
☐عوامی سیٹ اپ ☐حکومت کا سیٹ اپ ☐نجی سیٹ اپ<br>☐کوئی نہیں	آپ نے کہاں سے ٹیکہ لگایا؟
**What symptoms did you observe after first dose of vaccine?**	☐ Swelling ☐ Redness ☐Itching<br>☐Fever ☐Headache ☐Muscle pain<br>☐Tiredness ☐Coughing ☐ Diarrhea<br>☐ Nausea and Vomiting ☐Breathlessness<br>☐joint pain ☐ Anaphylactic reaction<br>☐Tingling and numbness ☐Swollen lymph nodes
☐سوجن ☐لالی ☐ کھجلی ☐بخار ☐سر درد<br>☐ پٹھوں میں درد ☐ تھکاوٹ ☐کھانسی ☐اسہال<br>☐متلی اور الٹی ☐سانس لینے میں دشواری ☐جوڑوں<br>کا درد ☐اینفیلیکٹک رد عمل ☐ پنوں اور سوئیاں کا<br>احساس ☐سوجن لمف نوڈس	ویکسین کی پہلی خوراک کے بعد آپ نے کن علامات کا مشاہدہ کیا؟
**What symptoms did you observe after second dose of vaccine?**	☐ Swelling ☐Redness ☐Itching<br>☐Fever ☐Headache ☐Muscle pain<br>☐Tiredness ☐Coughing ☐ Diarrhea<br>☐ Nausea and Vomiting ☐Breathlessness<br>☐joint pain ☐ Anaphylactic reaction<br>☐Tingling and numbness ☐Swollen lymph nodes
☐سوجن ☐لالی ☐ کھجلی ☐بخار ☐سر درد<br>☐ پٹھوں میں در د☐ تھکاوٹ ☐کھانسی ☐اسہال<br>☐متلی اور الٹی ☐سانس لینے میں دشواری ☐جوڑوں کا<br>درد ☐اینفیلیکٹک رد عمل ☐پنوں اور سوئیاں کا احساس<br>☐سوجن لمف نوڈس	ویکسین کی دوسری خوراک کے بعد آپ نے کن علامات کا مشاہدہ<br>کیا؟
