# Supplementary material for: COVID-19 vaccines side effects among the general population during the pandemic: a cross-sectional study
Source: Front Public Health. 2025 Mar 6;13:1420291. doi: 10.3389/fpubh.2025.1420291 (PMC11922928; doi:10.3389/fpubh.2025.1420291)
Supplement: Supplementary file 2 [file Data_Sheet_2.PDF]

No. F.18(Gen)/ED/2020
National Institute of Health, Islamabad
Ministry of National Health Services, Regulations & Coordination
Phone: (92-051) 9255211 **Fax:** (92-051) 9255125 - **Email:** edofficenih@gmail.com
National Focal Point for International Health Regulations

27 July 2020

To Whom It May Concern

A joint research project titled "Treatment regimens for management of COVID19 patients and their effectiveness" is being carried out at National Institute of Health, Islamabad Pakistan in collaboration of EMPHNET. The project aims to collect data on treatment regimens used for management of COVID19 patients at corona isolation wards in your hospital. The objective is to assess various treatment regimens and correlate with the outcomes on disease in COVID19 affected patients.

Dr. Hashaam Akhtar is conducting the above-mentioned project. It is requested that permission may be granted to him for collection of data as per attached questionnaire.

Your cooperation will be highly appreciated.

Thank you.

Professor
Dr. Aamer Ikram, SI(M)
Executive Director

INSTITUTIONAL RESEARCH FORUM

RAWALPINDI MEDICAL UNIVERSITY, RAWALPINDI

CERTIFICATE OF ETHICAL & INSTITUTIONAL APPROVAL TO CONDUCT RESEARCH

Ref. No. 163/IREF\RMU\2020 Dated: 19-08-220

This is to certify that Institutional Research Forum has evaluated the Research Proposal of the applicant visitor researcher, **Dr. Hashaam Akhtar**, Country Representative, The Eastern Mediterranean Public Health Network (EMPHNET) Global Health Development (GHD). The title of his research project is **"TREATMENT REGIMENS USED FOR MANAGEMENT COVID-19 AND THEIR EFFECTIVENESS"**

The Institutional Research Forum grants approval of his research on following conditions:

I. The applicant researchers abide by Rawalpindi Medical University Code of Practice for Research, alongside ethical guidelines.

II. Any changes affecting the ethical aspects of the project, any adverse or unseen events that occur will be reported to the relevant Ethics Committee of Rawalpindi Medical University and Institutional Research Forum through Research Coordinator, Research Unit, Rawalpindi Medical University.
